# Evaluating knowledge-based security questions for fallback authentication

**DOI:** 10.7717/peerj-cs.903

**Published:** 2022-03-11

**Authors:** Reem AlHusain, Ali Alkhalifah

**Affiliations:** Department of Information Technology, College of Computer, Qassim University, Buraidah, Saudi Arabia

**Keywords:** Authentication, Knowledge-based authentication, Fallback Authentication, Secret questions

## Abstract

Failed user authentication is a common event. Forgotten passwords and fingerprint non-recognition are the most common causes. Therefore, there is a need for efficient backup authentication methods, known as fallback authentication. However, fallback authentication methods suffer from different security and usability issues. This study aims to improve the security and usability of knowledge-based fallback authentication in the form of static security questions. The approach proposed in this study was designed considering different factors, such as question features, authentication mechanisms, and the use of tools to aid in composing memorable and secure answers. This study used a two-part experiment with 23 participants to evaluate the proposed approach based on security model testing. The results show that the proposed approach offered improved resistance to blind guess, focused guess, and observation guess attacks. While usability was clearly improved with questions that were based on recognition mechanisms, our results indicate that fallback authentication systems need a flexible level of security and avoidance of complexity in composing answers. In addition, our results indicate the effectiveness of using user behavioral details in the choice of topics for questions, where behavioral questions must have both high recall levels and resistance against guessing attacks. This work theoretically extends the knowledge of fallback authentication research by evaluating new security questions for fallback authentication considering replace of classical topics of security questions by introducing new topics of security questions based on user behavior and personal preferences. Also, this study applies methods of managing answers to security questions by encouraging users to compose answers based on free strict rules that inspire them to create strong and memorable answers based on their own rules. In addition, the findings of this study could support the deployment of knowledge-based authentication in fallback systems as a practical contribution to the user authentication field.

## Introduction

User multi-factor authentication plays a critical role in accessing online services. However, managing online identity represents one of the main challenges of digital transactions for a variety of reasons, such as the increased number of online identities for each user, limited user memory, security breaches, poor user habits, and failed authentication (Bonneau et al., 2012; Woods & Siponen, 2018). In 2021, the global market for multi-factor authentication is estimated to rise from USD 11.1 billion to USD 23.5 billion by 2026 (Marketsandmarkets, 2021d). Therefore, these challenges and economic predictions increase the need for additional efforts to explore user authentication and related issues.

Previous studies indicate a significant proportion of cybersecurity breaches are caused by individuals who do not comply with security policies (Alshaikh, 2020). According to recent security reports, mitigating data breaches can be achieved by considering human factor in the security improvement process (IBM, 2020; SANS, 2021). While (23%) of data breaches were caused by human error, and (19%) of the data breach was caused due to stolen or compromised credentials (IBM, 2020). One of the aspects of human error vulnerability is the difficulties of managing knowledge-based authentication by end-users, which leads to undesirable behavior (Yildirim, 2017; Michalíková, 2020). The security vulnerability in knowledge-based authentication is mainly due to the user behavior and practices, not related to the authentication system itself (Yildirim, 2017). According to the password malpractice report by Keeper Security (Keepersecurity, 2021), 57% of workers write their passwords on sticky notes and 49% of workers save passwords in unprotected plain-text documents. Therefore, previous studies highlight the importance of including human factors in security research, as it represents important aspects of computer security (Yildirim, 2017; Keepersecurity, 2021; Furnell, 2014). Previous studies show that the main reason for undesirable user behavior in managing authentication data is that end users have difficulties in understanding security policy and instructions properly (Yildirim, 2017; Furnell, 2014). While there is a lack of sufficient clarification to help users understand security rules in creating authentication data in a knowledge-based method, user guidance by clear security rules will aid users in composing memorable and strong authentication secrets (Yıldırım & Mackie, 2019; Furnell, 2014).

Authentication systems contain two main mechanisms, recall, and recognition, that depend on different ways of retrieving information from human memory. The recall mechanism is considered harder than the recognition mechanism as it depends on retrieving the right authentication secret from memory, such as passwords and answers to security questions. The recognition mechanism is considered easier as the user needs to identify whether the information provided is correct or not (Nielsen Norman Group, 2021).

Fallback authentication methods include security questions and reset links sent by email or SMS. Security questions can be of either static or dynamic type (Albayram & M. Khan, 2016). Static security questions are predefined questions that the user answers during registration, while dynamic security questions are generated based on the user’s activity data as captured by smartphones, such as mobile usage data and visited locations (Albayram & M. Khan, 2016; Addas, Salehi-Abari & Thorpe , 2019). With fallback authentication using security questions, if the question is static, the answer must match the predefined answer, while the answers to the dynamic security questions must match the information collected about the user. The other type of fallback authentication, reset links sent by email or SMS, works by preset recovery email addresses and phone numbers. However, recovery emails and phone numbers remain target points for attacks because authentication depends on the level of security offered by email and communication channels which may be inadequate. In addition, all fallback authentication methods suffer from different security and usability issues, such as vulnerability to guessing attacks, which remain a common form of security attack (Bonneau et al., 2012), and the memorability of fallback authentication data as a usability challenge (AlHusain & Alkhalifah, 2021).

This study aims to improve the security and usability of fallback authentication by focusing on static security questions. Static security questions were selected first because most of the recent studies conducted on fallback authentication have focused on dynamic security questions (Albayram & M. Khan, 2016; Addas, Salehi-Abari & Thorpe , 2019; Anani & Ouda, 2017; Hang et al., 2015a; AlHusain & Alkhalifah, 2021; Anvari, Pan & Zheng, 2020; Ebrahim & Vadakkumcheril, 2019; Albayram et al., 2015) with little attention paid to improving static security questions (Micallef & N. Arachchilage, 2021; Bonneau et al., 2015; Schechter, A. Brush & Egelman, 2009; Schechter & Reeder, 2009). However, this limited used mechanism of static questions as fallback authentication has some limitations. For instance, studies (Schechter, A. Brush & Egelman, 2009; Schechter & Reeder, 2009) found security questions could be difficult for the user to answer and easy for the attackers to guess. They revealed that the answers to challenge questions may be observable, and it is easy for an attacker to detect or recover answers to challenge questions (Schechter, A. Brush & Egelman, 2009; Schechter & Reeder, 2009). However, a previous study (Bonneau et al., 2015) found that security questions can still be a useful lightweight authentication method, provided the risk level is considered low. Therefore, the study (Bonneau et al., 2015) concludes by open question to find more identity confirmation questions that are both secure and easy to answer. The second reason is that passwords are still the most common primary authentication method (Quermann, Harbach & Dürmuth, 2018). Our study argues that security questions could also be used as a secondary authentication method (fallback method) with improvements comparable to previously conducted efforts with passwords (Bonneau et al., 2015; Schechter, A. Brush & Egelman, 2009; Schechter & Reeder, 2009). Therefore, this study introduces a new design by improving the security and the usability of the static questions as a fallback authentication approach.

The security and usability of static security questions were investigated in this study through a two-part experiment conducted with 23 participants. The study planned to explore three different objectives. First, the study explored the features of the question themselves that lead to increased security question performance by examining the effects of different factors such as question form, type of recall mechanism, and the content of the questions. Second, the study explored the effect of using supporting methods and following user guidance to compose memorable and secure answers. Finally, the study investigated the effects of the demographic details of the participants (gender, technical background, educational level, and method of creating and saving knowledge-based authentication data). The study results indicate that the proposed approach increases resistance to guessing attacks by increasing the strength of answers. In addition, the study offered an acceptance usability level in questions in the form of a recognition-based authentication mechanism.

Deploy-ability of security questions as a part of multifactor authentication is strong and remains the first choice to apply multifactor authentication in many organizations (Okta, 2021). Therefore, this study contributes to improving security and usability of security questions by considering the following points:

Firstly, replace the classical topics of security questions (*e.g.*, what is your date of birth?) by introducing new topics of user behavior and personal preferences.

Secondly, explore the factors that affect the performance of security questions using a comprehensive approach that includes the features of security question themselves, as well as authentication mechanisms.

Thirdly, apply the methods of managing answers to security questions by encouraging users to compose answers based on free strict rules that inspire them to create strong and memorable answers based on their own rules.

**Figure 1:**
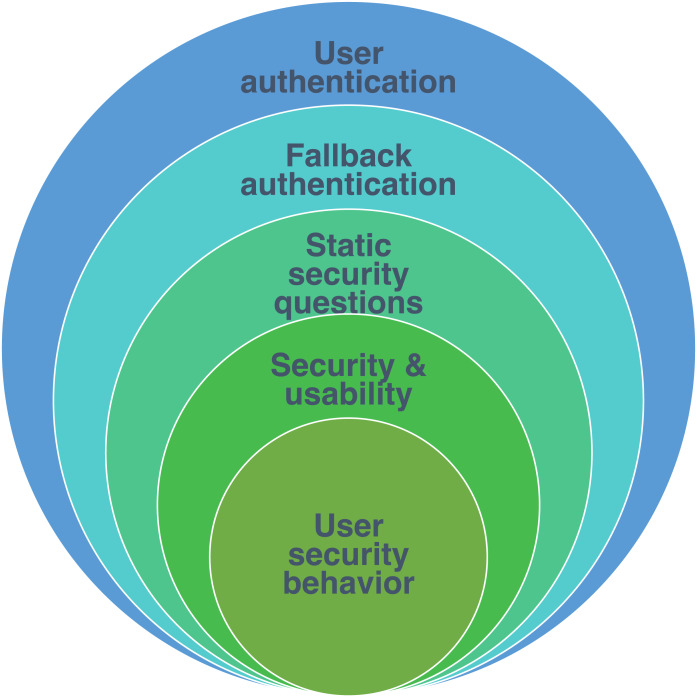
Study scope.

Therefore, the main context of this study is user authentication. It considers specifically the fallback authentication in the mechanism of static security questions. This study aims to enhance only the security and usability features. In addition, it is concerned with investigating issues of user security behavior as shown in Fig. 1.

## Related Work

Several studies have explored fallback authentication in dynamic security questions by collecting information related to users’ activities, such as geographical information, social information, and mobile usage data. Addas, Salehi-Abari & Thorpe (2019) proposed a location-based fallback authentication system by developing an application for geographical security questions (GeoSQ). The proposed solution works by utilizing autobiographical location data for generating security questions such as “Where were you on the 14th of February at 4:00 PM?”. The user can then respond to the location-based question by navigating to Google Maps and setting a marker on the correct location. The proposed system was designed by asking users 10 questions; successful authentication conditions include accuracy of the location within 200 m, and the user correctly answering seven out of 10 of the location questions. Through a user study, the authors analyzed the security and usability of the proposed system. The security of the design was analyzed by testing the resilience of the GeoSQ system against various security threats, such as throttled online guessing attacks, known adversary attacks, and phishing. The study obtained an acceptable level of security for the proposed system. However, for usability, the authors decided that the proposed system required improvements to increase user memorability for the logged locations.

Siddavatam et al. (2019) proposed a fallback authentication system based on dynamic security questions related to the user’s information from different social networking sites. The proposed system works by using social information data that the user has uploaded over time to social media sites; based on the collected data, the system generates security questions that vary in degrees of difficulty.

Albayram & M. Khan (2016) explored fallback authentication in dynamic security questions based on users’ smartphone usage data, with questions such as “Who called you at <time>?” The study aimed to mitigate the limitations of fallback authentication mechanisms based on static challenges that are known to be easy to predict. Their study revealed that the style of challenge questions and the format of responses have a major impact on user results. In the same context, Hang et al. (2015b) proposed a fallback authentication system that works by generating security questions based on mobile usage data. The system generates security questions related to apps either installed or not installed on a user’s phone (*e.g.*, “Is this app installed on your device?”); the study aimed to help users who may face difficulties in using current fallback authentication methods. Thus, this study highlights the importance to consider a wider variety of users when improving current fallback authentication systems.

In addition, efforts have been made to improve the usability of knowledge-based authentication, such as increasing the recall level of authentication information–e.g., answers to questions or passwords. For example, free strict rules can have a positive effect on the recall of knowledge-based authentication. Yıldırım & Mackie (2019) performed a study based on the use of free strict rules for passwords. The approach works by helping users to create their methods and rules in composing strong and memorable passwords. The authors argue that using a free strict rules approach increases user compliance with security rules. Supporting this point, Furnell (2014) showed in his study that most websites lack sufficient clarification to help users understand the importance of following the security rules. They suggested that involving rational factors in security rules would increase user awareness of the security of authentication information.

Other studies have been directed toward improving the usability of authentication systems through graphical features, such as that of Karim, Shukur & AL-banna (2020), who proposed a novel fallback authentication system known as user interface preferences authentication (UIPA). The proposed system works based on user interface (UI) preferences. The authors selected this method to improve the effectiveness of a traditional approach to fallback authentication. This study showed the importance of involving the user interaction feature with an authentication system, which refers to the human–computer interaction (HCI) field and highlights the role of the design factor in the authentication process. The study evaluated the proposed system by system efficiency and system acceptance (technology acceptance model [TAM]), which demonstrated the high efficiency of the proposed system. In addition, the study showed that the users were satisfied with and able to adopt such a system.

These efforts inspired us to contribute to the improvement of static security questions by investigating the effect of question features and authentication mechanisms. This study explores the effect of using free strict rules on static security questions. In addition, it investigates the effect of participants’ demographic details.

## Methodology

**Figure 2:**
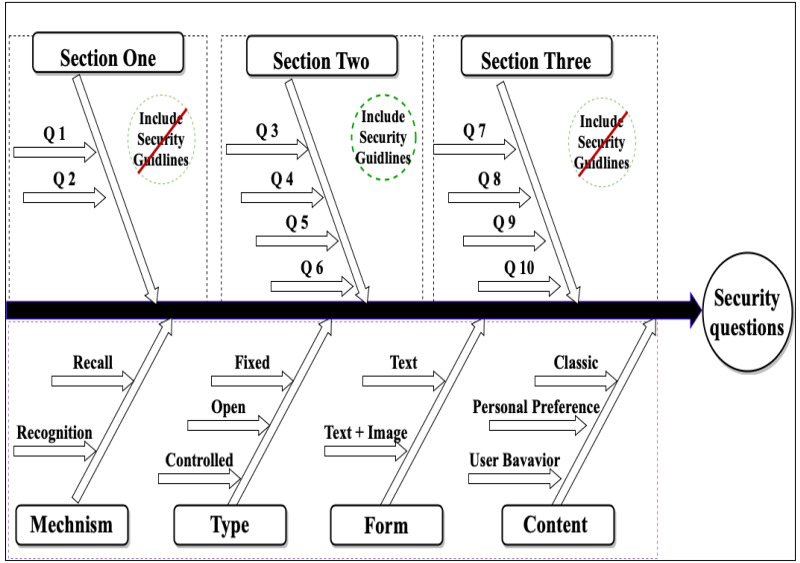
Overview of the security questions across the examined features.

This work aims to improve static security questions in security and usability perspectives through an online experimental method. Therefore, we designed a fallback authentication application based on static security questions using a Google Apps Script. The fallback application contains three sections of ten questions with different features, as shown in Fig. 2, Tables 1 and 2. The questions’ features were created based on question types described by Just (2004). Security questions based on user behavior were designed using implicit memory, which depends on the unintentional recollection of information; this type of memory used in authentication systems helps users easily recall their authentication information (Yang et al., 2020; Castelluccia et al., 2017). According to previous studies (Crawford, Godbey & Crouter, 1986; Kuder, 1939), personal preferences for information can remain stable for a long time; therefore, creating security questions based on this information is possible. The goal of the first section of the experiment was to measure the user memorability of the questions. Section two was designed to measure the effect of tools to aid in composing strong and memorable answers using two suggested methods: abbreviations and meaningful answers. The abbreviations method consists of selecting an abbreviation to help the user remember their answers, as shown in Table 3; the meaningful answer method is based on composing a descriptive answer. These methods have been discussed in other studies (Yıldırım & Mackie, 2019; Woo & Mirkovic, 2016). Section three was designed to measure the effect of applying the recognition mechanism.

**Table 1:** Overview of the security questions used in study experiment.

**Section number**	**Questions of the section**
**Section one**	Q1.1 From the list below, select your favorite class in high school Q1.2 What was your teacher’s name of this course?
	Q2 Your dream job or business is ______?
**Section two**	Q3 After an achievement in your work, name the favorite vacation place?
	Q4 Write a description of the the image below
	Q5 What is your favorite mobile applications?
	Q6 Select an English words that you usually pronounced correctly?
**Section three**	Q7 From the list below, select the image that reflects your hobby
	Q8 IF Your national ID Expiration date is in this year, in which of the available options you may select to renew it?
	Q9 What was the color of your first mobile phone?
	Q10 In case you invited to attend a formal event, what the most thing you will think of?

**Table 2:** Overview of the security questions across the examined features.

**Question features**	**Sub-features**	**Q1**	**Q2**	**Q3**	**Q4**	**Q5**	**Q6**	**Q7**	**Q8**	**Q9**	**Q10**
**Type**	**Fixed**	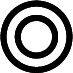	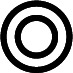	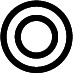	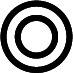	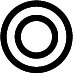	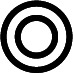	•	•	•	•
	**Open**	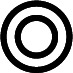	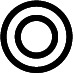	•	•	•	•	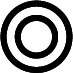	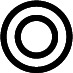	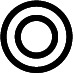	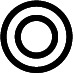
	**Controlled**	•	•	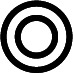	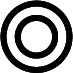	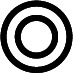	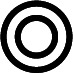	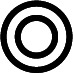	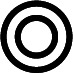	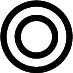	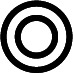
**Form**	**Text only**	•	•	•	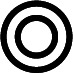	•	•	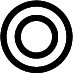	•	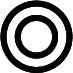	•
	**Text & image**	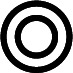	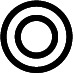	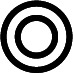	•	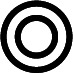	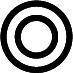	•	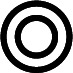	•	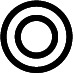
**Content type**	**Classic**	•	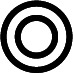	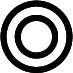	•	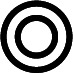	•	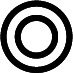	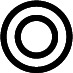	•	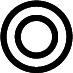
	**Personal preference**	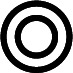	•	•	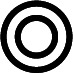	•	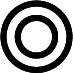	•	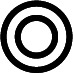	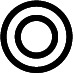	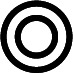
	**User behavior**	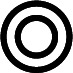	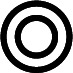	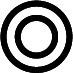	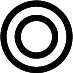	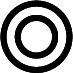	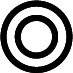	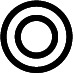	•	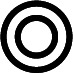	•
**Authentication mechanism**	**Recall**	•	•	•	•	•	•	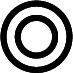	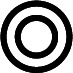	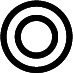	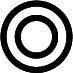
	**Recognition**	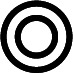	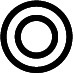	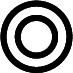	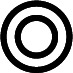	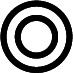	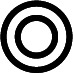	•	•	•	•
**User guidance**	**Requirements and examples**	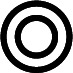	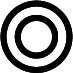	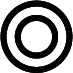	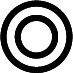	•	•	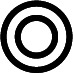	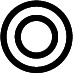	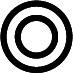	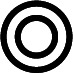
	**Using aid tools of memorability**	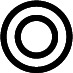	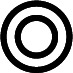	•	•	•	•	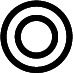	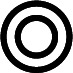	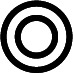	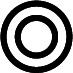

**Table 3:** Method of abbreviations (Yıldırım & Mackie, 2019).

**Aid tools of memorability**	**User rule steps**	**Example to answer** **Q.** Your dream job or business is ______?
**Method of abbreviations**	**Step 1: select answer to question**	have my own business
	**Step 2: letters selection (capitalize or small) *e.g.* user select first letter of each word is a capital.**	Have My Own Business
	**Step 3: Numbers and symbols selection *e.g.* answer start by this value 510@&**	510@& Have My Own Business
	**Step 4: select answer abbreviation** (users can even write the abbreviation somewhere to help them remember it. As long as, no one knows user formula that converts the abbreviation into a strong answer, abbreviation is meaningless to them.)	*e.g.* HMOB (user select first letter of each word as a hint to their answer)
**Result**	**The answer to the security question:** **510@&** Have My Own Business	Abbreviation HMOB

The experiment survey was structured to be multilingual (English and Arabic) so that it would be accessible by all participants. The link for the survey was available online to participants to be filled out at any time. The time spent on each question was recorded to measure usability features.

This study aims to examine the performance of the proposed security questions through a two-part experiment by 23 participants. This number of participants can be considered acceptable. For example, a study (Nngroup, 2022) indicated that the required number of participants involving in a binary metric of (success, conversion) is 21 for low risk, fair precision with 95% confidence level and 20% of desired margin of error (Nngroup, 2022). Another study suggests that the minimum number of sample size in user experience studies that use questions that are continuous or multipoint scale such as usability and security metrics is 20 (Sauro, 2016). Previous studies on KBA and evaluating security questions also used small-sample experiments involving 20 (Micallef & N. Arachchilage, 2017) and 15 (Albayram et al., 2015) participants.

An informed consent was obtained from each participant *via* email. Each participant was given an option to choose to participate in the experiments by accepting the invitation; otherwise, they could decline or ignore the invitation to participate. The participation was completely voluntary, and no compensation was given to the participants. Therefore, the non-probability sampling technique was selected by convenience sampling of participants including colleagues, and friends. The non-probability sampling was selected due to three main reasons. Firstly, as the nature of our study is exploratory research to quantify the problems of security questions, this study needs a particular sample not a random selecting of participants. Secondly, to ensure respondents respond quickly to complete the second experiment. Thirdly, include a sample of participants that involve a relationship with each other (*e.g.*, partners or best friends) to test the security level of the proposed security questions.

**Demographic details:** The ages of the recruited participants ranged from 18 to 34 years. Out of 23 participants, 14 were female (61%). Most participants (70%) possessed a B.C. degree. The majority of the participants had a technical background. The memorize-only and written-down methods were used by participants most often in saving knowledge-based authentication data. In addition, the majority of participants (61%) included specific names in creating their knowledge authentication data. Table 4 describes the demographic details of the participants.

**Table 4:** Demographics details of the participants.

**Demographics details**	**Result**
**Gender**	
Female	14
Male	9
Grand Total	**23**
**Education**	
B.C	16
MS	4
High School	2
diploma	1
Grand Total	**23**
**Technical background**	
Medium Background	14
High Background or specialist	8
No background	1
Grand Total	**23**
**Method of creating knowledge-based authentication data**	
Includes specific names	14
Includes specific dates *e.g.* birthdate	4
In randomly way	3
Includes complex words	2
Grand Total	**23**
**Method of saving knowledge-based authentication data**	
By written down	9
By memorize only	9
By Automatic login	3
By memorize and use helping methods	2
Grand Total	**23**

## Security and Usability Analysis

The security and usability of the proposed fallback authentication were evaluated by conducting an online user study (*n* = 23). The user study consisted of two parts: the first part involved users composing answers to the security questions, while the second part was an experiment performed within 15 days after the first part to measure the memorability of the users’ answers. In this section, the security and usability evaluations are presented sequentially.

### Security analysis

In order to analyze the answers to the questions, previous studies used testing methods to measure knowledge-based authentication data in security questions. Knowledge (answer) strength can be calculated by entropy formula based on Shannon information theory (Shannon, 1948) according to Just and Aspinall (Just & Aspinall, 2009). The entropy level is used as a stander of representing the strength of the knowledge-based authentication such as passwords according to the National Institute of Standards and Technology (NIST) (Grassi, Garcia & Fenton, 2017).

The goal of calculating answer entropy is to specify the difficulty of predicting an answer. Answer entropy represents how strong an answer is, the three levels of which are high, medium, and low. The security testing of the study was built on the security model testing proposed by Just & Aspinall (2009). We classified security testing into two groups according to the answer type: answers in the form of open text and answers in the form of a fixed response.

Answers in the form of open text (Q1–Q6) were compatible with measurement by answer entropy using the following formula (Shannon, 1948; Taveras & Hernandez, 2018):

\begin{eqnarray*}\mathbi{E}={\log \nolimits }_{2}{\mathbi{R}}^{\mathbi{L}} \end{eqnarray*}

where E = answer entropy in bits; R = pool of characters (lowercase letters 26, upper case letters 26, numbers 10, special characters 33); L = length of answer.

The security testing levels were calculated as shown in Table 5 (Just & Aspinall, 2009) and presented in Table 6 (Khan, 2011).

**Table 5:** Levels of answer entropy (Just & Aspinall, 2009).

Low	less than 2^34^ possible answers
Medium	between 2^34^ and 2^48^ possible answers
High	greater than 2^48^ answers

**Table 6:** Answer strength to questions in form open question (Q1–Q6).

**Question number**	**Security analysis**				
	**Answer length** **(characters)**		**Answer strength**		
	Mean*	SD* *σ*	**High** **%**	**Medium** **%**	**Low** **%**
Q1	8.65	6.01	39.13% (that mean Q1 contains 9 answers out of 23 is in high strength)	4.34%	56.52%
Q2	18.73	12.36	82.60%	0%	17.39%
Q3	13.56	13.63	47.82%	8.69%	43.47%
Q4	20.60	14.41	82.60%	8.69%	8.69%
Q5	14.26	8.55	78.26%	13.04%	8.69%
Q6	16.82	8.87	91.30%	0%	8.69%

**Notes.**

* Mean is average*SD the standard deviation (Khan, 2011) is “a measure of the amount of variation or dispersion of a set of values” (23 answers to each question). “A low standard deviation indicates that the values tend to be close to the mean (also called the expected value) of the set, while a high standard deviation indicates that the values are spread out over a wider range”.

Entropy values represent the guess-ability levels of answers. Blind guess and focused guess models (Just & Aspinall, 2009) were applied to Q1–Q6. Table 7 shows the probability of blind guesses and focused guesses for each answer based on calculating knowledge entropy in bits; a high value indicates a weak answer that can be guessed easily, and a low value means the answer is strong and difficult to guess.

**Table 7:** Blind Guess and Focused Guess models on recall-based questions (Q1–Q6).

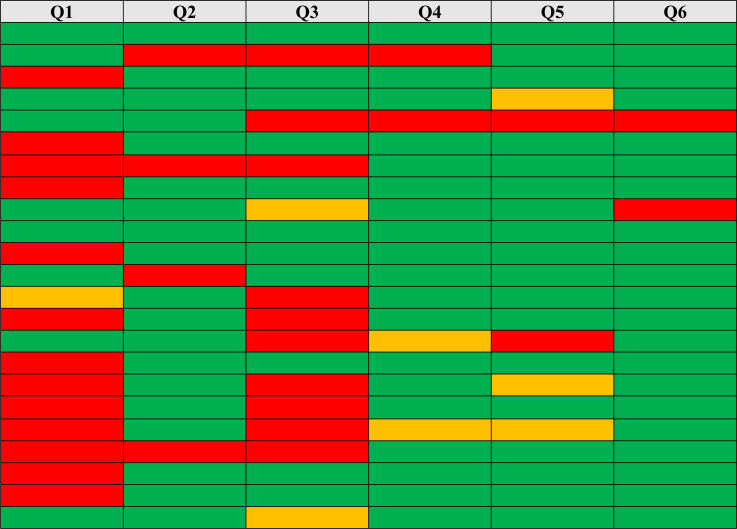

**Notes.**

*Where probability of guessing is either high (red), medium (yellow), or low (green).

The second security testing was performed with the observation guess model (Just & Aspinall, 2009) by close adversaries on the questions of recognition mechanism (Q7–Q10), as shown in Table 8. Close adversaries are defined as adversaries who have prior knowledge about the legitimate user. Observation guessing was conducted with a select group of study participants (11 pairs) with a close relationship with each other (*e.g.*, partners or best friends), as shown in Table 9. In each pair, one person acted as the close adversary and the other person acted as the legitimate user. As the Table 8 shows the number of correct attempts in guessing the answer to each question.

**Table 8:** Overview of Observation Guess to questions in form of recognition mechanism (Q7–Q10).

**Q#**	**# of correct answers by** **close adversaries**	**# of answers neither guessed by close adversaries**	**Total**
**Q7**	4	7	11
**Q8**	3	8	11
**Q9**	5	6	11
**Q10**	5	6	11

**Table 9:** Relationships of participants in Observation guessing attack.

Observation guesting attack participants	Relationship	
	Friendship	Family members
1	**✓**	
2		**✓**
3	**✓**	
4		**✓**
5		**✓**
6		**✓**
7		**✓**
8		**✓**
9		**✓**
10		**✓**
11		**✓**

### Usability analysis

**Figure 3:**
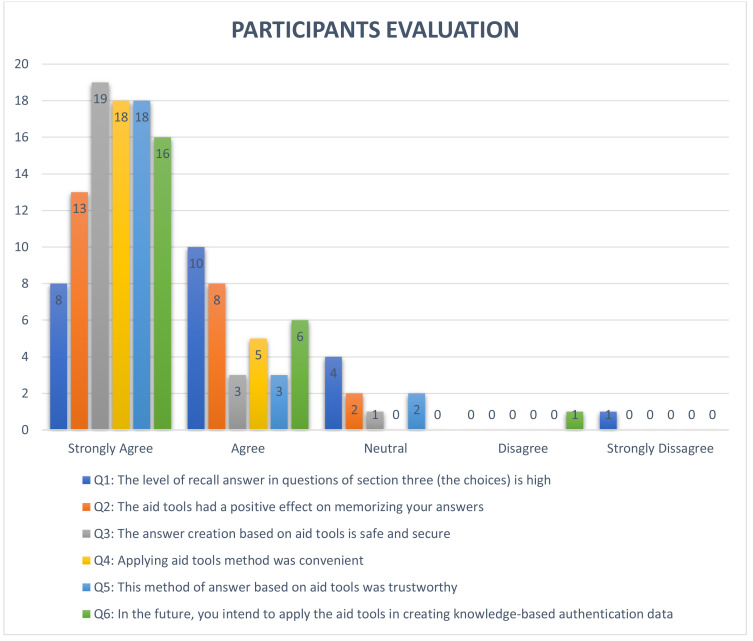
Result of evaluation by participants of the study.

One of the most important features for security questions is the memorability level, which represents the ease with which the user recalls answers. In order to test this feature, we first calculated the number of correct answers by using an exact equation that compares two text strings (answer1, answer2) and returns TRUE if they are exactly the same, and FALSE otherwise (Microsoft, 2021), as shown in Table 10. The second-string metric used in this study was the Levenshtein function (Levenshtein, 1966) to measure the difference between user answers (answer1, answer2) which were submitted in Experiment 1 and Experiment 2. The Levenshtein function is defined as the minimum number of editing operations (deletion, insertion, and substitution) needed to convert string A to string B (for example), as shown in Table 11 (Ma et al., 2010). The Levenshtein results for the security questions are shown in Table 12. This metric of editing distance is used in different fields to measure changes to any string, such as in cryptography, where it is used to measure the confidentiality of a message in order to support privacy features (Rane & Sun, 2010). To measure the user acceptance level of the proposed approach, the participants of the study answered a series of questions related to their user experience at the end of the experiments to determine the participants’ opinions of the proposed techniques. Overall, participants of the study were satisfied with the proposed approach, as shown in Fig. 3. Finally, the overall usability of all security questions in the proposed approach was analyzed based on completion time and memorability levels, as shown in Table 13 (Just & Aspinall, 2010).

**Table 10:** Overview of EXACT function result.

**Q1**	**Q1.2**	**Q2**	**Q3**	**Q4**	**Q5**	**Q6**	**Q7**	**Q8**	**Q9**	**Q10**
TRUE	FALSE	TRUE	FALSE	FALSE	FALSE	FALSE	TRUE	FALSE	TRUE	TRUE
TRUE	TRUE	FALSE	FALSE	FALSE	FALSE	FALSE	TRUE	TRUE	TRUE	TRUE
TRUE	TRUE	FALSE	TRUE	FALSE	FALSE	FALSE	TRUE	TRUE	TRUE	TRUE
TRUE	FALSE	FALSE	FALSE	FALSE	FALSE	FALSE	FALSE	FALSE	TRUE	TRUE
TRUE	TRUE	FALSE	TRUE	FALSE	FALSE	TRUE	TRUE	TRUE	FALSE	TRUE
TRUE	TRUE	FALSE	FALSE	FALSE	FALSE	FALSE	TRUE	FALSE	FALSE	TRUE
TRUE	TRUE	FALSE	FALSE	FALSE	FALSE	FALSE	TRUE	TRUE	FALSE	TRUE
TRUE	TRUE	FALSE	FALSE	TRUE	TRUE	TRUE	TRUE	TRUE	TRUE	TRUE
TRUE	TRUE	TRUE	FALSE	FALSE	FALSE	FALSE	TRUE	TRUE	FALSE	TRUE
TRUE	TRUE	FALSE	FALSE	TRUE	TRUE	TRUE	TRUE	TRUE	TRUE	TRUE
TRUE	FALSE	FALSE	FALSE	FALSE	FALSE	FALSE	TRUE	TRUE	FALSE	TRUE
FALSE	FALSE	FALSE	FALSE	FALSE	FALSE	FALSE	FALSE	FALSE	FALSE	TRUE
TRUE	TRUE	TRUE	TRUE	TRUE	FALSE	FALSE	TRUE	TRUE	TRUE	TRUE
TRUE	TRUE	FALSE	TRUE	FALSE	FALSE	FALSE	TRUE	TRUE	TRUE	TRUE
TRUE	TRUE	TRUE	FALSE	FALSE	FALSE	FALSE	TRUE	TRUE	TRUE	TRUE
TRUE	FALSE	FALSE	TRUE	FALSE	TRUE	TRUE	FALSE	TRUE	TRUE	TRUE
TRUE	FALSE	FALSE	FALSE	FALSE	FALSE	FALSE	TRUE	TRUE	TRUE	TRUE
TRUE	FALSE	FALSE	FALSE	FALSE	FALSE	FALSE	TRUE	TRUE	TRUE	TRUE
FALSE	FALSE	FALSE	FALSE	FALSE	FALSE	FALSE	TRUE	FALSE	FALSE	TRUE
FALSE	FALSE	TRUE	FALSE	FALSE	FALSE	FALSE	TRUE	TRUE	TRUE	FALSE
FALSE	FALSE	FALSE	FALSE	FALSE	FALSE	FALSE	TRUE	TRUE	TRUE	TRUE
FALSE	FALSE	FALSE	FALSE	FALSE	FALSE	FALSE	FALSE	FALSE	FALSE	TRUE
TRUE	FALSE	TRUE	FALSE	FALSE	FALSE	FALSE	TRUE	TRUE	FALSE	FALSE

**Table 11:** Explanation to the work of Levenshtein editing distance by examples.

**String A**	**String B**	**Levenshtein editing distance**
zoe	coe	1
the	zoe	2
The	the	1
The	The	0

**Table 12:** Levenshtein editing distance result on security questions of the study.

**#**	**Q1**	**Q1.2**	**Q2**	**Q3**	**Q4**	**Q5**	**Q6**	**Q7**	**Q8**	**Q9**	**Q10**
**1**	0	2	0	5	23	1	10	0	2	0	0
**2**	0	0	4	4	5	5	5	0	0	0	0
**3**	0	0	8	0	1	1	11	0	0	0	0
**4**	0	5	24	20	17	19	23	1	6	0	0
**5**	0	0	1	0	3	9	0	0	0	6	0
**6**	0	0	1	52	26	17	16	0	2	3	0
**7**	0	0	1	5	6	4	7	0	0	4	0
**8**	0	0	3	11	0	0	0	0	0	0	0
**9**	0	0	0	7	9	10	14	0	0	6	0
**10**	0	0	3	11	0	0	0	0	0	0	0
**11**	0	11	5	32	7	12	22	0	0	6	0
**12**	8	4	1	4	28	7	15	2	2	6	0
**13**	0	0	0	0	0	4	5	0	0	0	0
**14**	0	0	30	0	2	13	11	0	0	0	0
**15**	0	0	0	16	9	7	13	0	0	0	0
**16**	0	1	3	0	3	0	0	1	0	0	0
**17**	0	7	4	21	1	12	6	0	0	0	0
**18**	0	2	15	11	14	13	6	0	0	0	0
**19**	7	7	43	1	45	11	24	0	2	6	0
**20**	22	5	0	5	7	27	19	0	0	0	28
**21**	5	7	7	5	24	20	10	0	0	0	0
**22**	7	10	18	18	17	4	9	1	6	6	0
**23**	0	18	0	18	27	3	10	0	0	6	28
**Max**	22	18	43	52	45	27	24	2	6	6	28
**Mean**	2.13	3.43	7.43	10.70	11.91	8.65	10.26	0.22	0.87	2.13	2.43
**SD *σ***	5.07	4.75	11.29	12.41	12.01	7.28	7.29	0.52	1.79	2.80	8.07

**Table 13:** Overview of usability evaluation based on completion time and memorability levels.

**Questions**		Completion time (In Seconds)		Match answer *					
				**Memorable**		**Repeatable**		**Not memorable**	
		**Mean**	**SD**	**%**	**#**	**%**	**#**	**%**	**#**
Q1	Q1.1	33.48	39.17	78.26%	18/23	***0%***	0/23	21.74%	5/23
	Q1.2			47.83%	11/23	39.13%	9/23	***13.04%***	3/23
Q2		32.89	55.56	26.09%	6/23	**47.83%**	11/23	26.09%	6/23
Q3		37.46	27.95	21.74%	5/23	**47.83%**	11/23	30.43%	7/23
Q4		42.91	25.15	***13.04%***	3/23	43.48%	10/23	**43.48%**	10/23
Q5		45.37	**87.19**	***13.04%***	3/23	43.48%	10/23	**43.48%**	10/23
Q6		64.03	79.39	17.39%	4/23	39.13%	9/23	**43.48%**	10/23
Q7		30.13	15.35	82.61%	19/23	***0%***	0/23	17.39%	4/23
Q8		16.11	9.68	73.91%	17/23	***0%***	0/23	26.09%	6/23
Q9		***12.62***	***7.93***	60.87%	14/23	***0%***	0/23	39.13%	9/23
Q10		**110.88**	55.88	**91.30%**	21/23	***0%***	0/23	8.70%	2/23

**Notes.**

* Memorable: EXACT same answer? Repeatable: Answer misspelled?, Not memorable: Different answer? (Just & Aspinall, 2010).

*Mean is average.

*SD the standard deviation.

** The higher value of a column is indicated by bold, and the underline indicates the lower value.

## Discussion

The proposed fallback authentication approach in this study offered improvements in the security and usability of static security questions when compared to traditional security questions. However, the features of the questions, such as authentication mechanism and content type, had an effect on the results. Questions based on the recognition mechanism produced high levels of recall, where approximately (60%–91%) of participants were able to correctly answer questions of section three (Q7–Q10), reached the highest value of (91%) in Q10, and reached their lowest value of (60%) in Q9 as shown in Table 13. In addition, questions based on the recall mechanism and in topics of classic content (Q1 & Q4 & Q6) offered low editing distance errors compared to personal preference questions (Q2 & Q3 & Q5) as shown in Table 12.

With regards to the content of the question, behavioral questions offered the best results in Q10 compared to Q8, where approximately (91%) of participants were able to correctly answer the Q10, compared to (73%) in Q8. Therefore, we noticed the effectiveness of using questions with topics relevant to user behavior and decisions, especially if the question is based on a recognition mechanism.

On the other hand, this study showed semantic and lexical ambiguity (Hang, 2016) in some of the answers, wherein the meaning was true, but the syntax was false. This lexical ambiguity was apparent in Q2, where 39% of the answers were semantically correct. The overview of answer error ambiguity is shown in Table 14.

**Table 14:** Ambiguity descriptions.

Ambiguity name	Type	Examples	
		Original answer	Wrong answer
Semantic ambiguity	Synonymous description *e.g.,*	Owner of business	Have my own business
	Prepositions	To Europe	Europe
	Long and short answer	Visit the Eastern Europe	Europe
	Punctuation marks *e.g.* (,) and (**.)**	Visit the Eastern Europe**.**	Visit the Eastern Europe**,**
	Different languages	Answer in English	Answer in Arabic
	Numbers writing	Two	2
Lexical ambiguity	uppercase letters in the first word	Application	application
	uppercase letters in each word	Application Developer	Application developer
	Whitespace	ApplicationDeveloper	Application Developer

**Figure 4:**
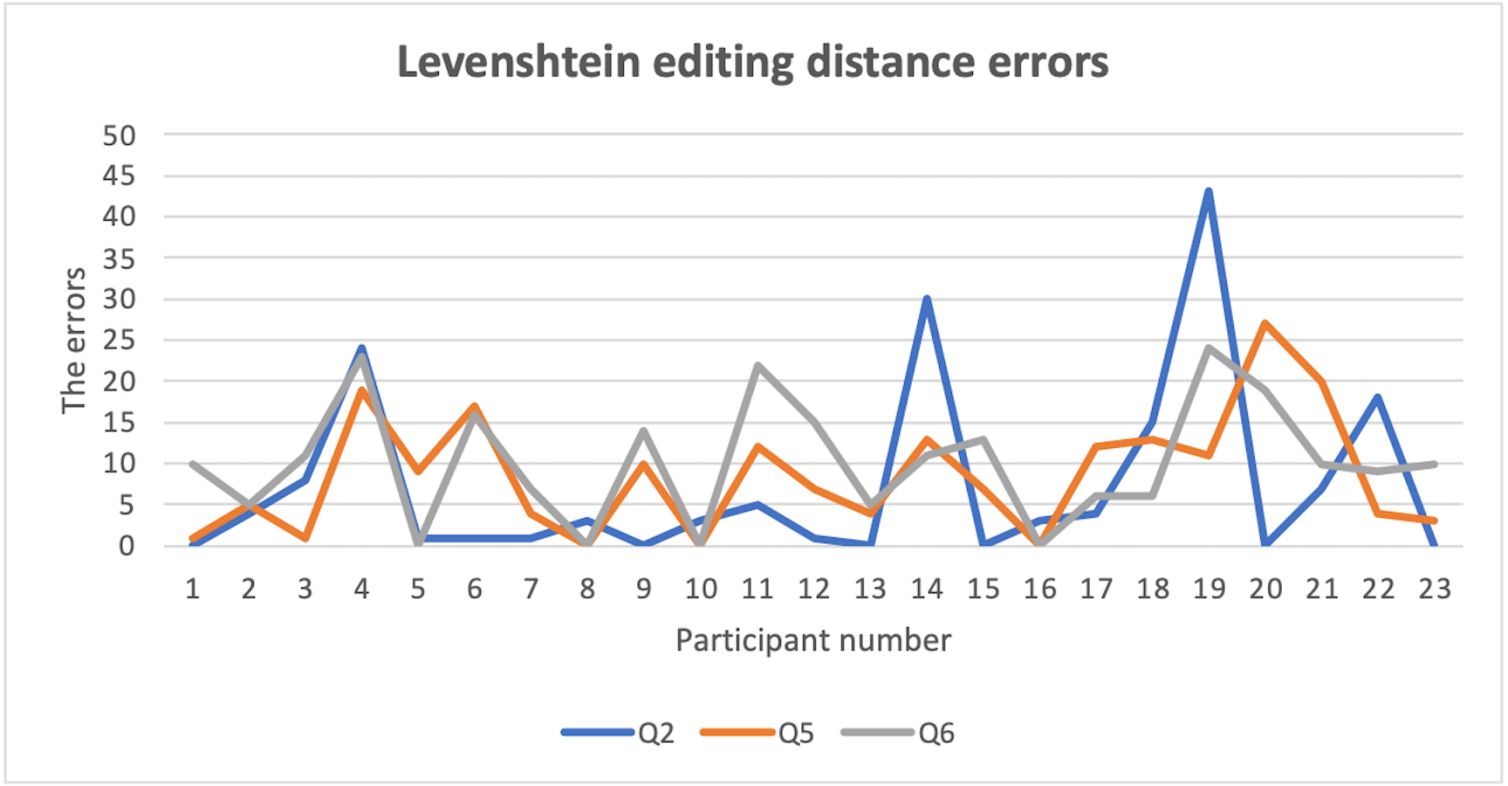
Levenshtein editing distance errors.

Open questions offered a high level of Levenshtein editing distance errors among users’ answers, as shown in Fig. 4, which is a normal result. The high error rate occurred due to the complexity of security requirements in these questions (section two Q3–Q6), which had a negative impact on users’ recall level of their answers.

The completion time of answering the security questions was shorter with the recognition mechanism in Q9, Q8, and Q7, followed by the recall mechanism of Q2 and Q1. Therefore, the recognition mechanism and question topics related to personal preference and user behavior had a positive effect on usability. The time spent to answer security questions in our study is reasonable compared to other studies (Markert et al., 2019; Addas, Salehi-Abari & Thorpe , 2019; Micallef & N. Arachchilage, 2017; Albayram et al., 2015) as shown in Table 15. Previous studies of static questions (Micallef & N. Arachchilage, 2017; Markert et al., 2019) recorded an average time between (5-16) minutes based on testing 3 questions only. Moreover, existing studies which evaluated dynamic questions, (Addas, Salehi-Abari & Thorpe , 2019; Albayram et al., 2015) recorded an average time between (2–5) minutes based on testing (1–10) questions, while our study accounts for 7 min as an average of time spent for all 10 questions.

**Table 15:** Time spent to answer security questions compared to previous study.

**Time Spent** **average** **(Time unit is in minutes)**	**Type of security question**	**Number of questions**	**Authentication mechanism**	**Participants**	**Duration**	**Study** **Reference**
16	Static	3	Recall	74	3 weeks	Markert et al. (2019)
5	Dynamic	10	Recognition	36	7–11 days after Session 1	Addas, Salehi-Abari & Thorpe (2019)
5	Static	3	Recall Recognition	20	1 day	Micallef & N. Arachchilage (2017)
2	Dynamic	1	Recognition	14–15	30 days	Albayram et al. (2015)
7	Static	10	Recall Recognition	**23**	**15 days**	**Our Study**

From Table 7, we noticed that using tools to assist users and following user guidance clearly raised the security level in questions of section 2, as demonstrated with the strong answers in Q5, with the approximate 18 out of 23 (78.26%) of answers is strong, and in Q6, approximate 21 out of 23 (91%) is strong answers.

Regarding resistance to guessing attacks, as described in Table 8. Questions in the topic of user behavior (Q8&Q10) offered the best result compared to questions in classic and personal preference (Q7&9). As shown in Table 8, Q8 contains only three correct answers by close adversaries compared to five correct answers in Q10. Therefore, we noticed that security questions were resistant to guessing attacks especially if the content of the question contained specific details that a user can recall, while others would not consider important, consequently the others were unable to guess the answer as in the case of Q8.

According to the study results related to the effects of participants’ demographic details, we found an unexpected result for the participants who use the method of saving knowledge-based authentication based on automatic login. Where they were able to answer five questions (Q1, Q3, Q8, Q9, Q10) 100% correctly, they accounted for a low Levenshtein editing distance error that did not exceed 11 characters.

Finally, security questions are applicable to use as part of multi-factor authentication. Since security questions are effective in two main features the cost and deployment, therefore organizations can utilize these features and add a layer of authentication to the fallback system. In addition, with security questions still in use by different service providers, such as Apple (Apple Support, (2022)), this type of research could change older versions of security questions. Therefore, security questions can be applied as part of a fallback authentication system supported by additional security measurements such as limited chances to answer and notify the user when this method is initiated.

## Conclusions

In this study, fallback authentication by static security questions was improved; there is still a research knowledge gap for this kind of security question. This work proposed to improve fallback authentication applications by considering different features such as question type, topic, and authentication mechanisms. In addition, the effects of using tools to aid in composing answers were explored. Through a user study (*n* = 23) evaluated with security model testing, the proposed approach offered high levels of strength in answers and resistance to the blind guess and focused guess attacks of 70%, while resistance to the observation guess attack was 61%. In addition, usability was improved, especially in questions with recognition mechanisms. Finally, we can conclude with future work recommendations. Security questions represent secondary authentication methods; therefore, a flexible level of security is important, such as the number of attempts allowed and avoiding complexity in composing answers. Error tolerance of knowledge-based fallback authentication systems should include consideration of these ambiguities in answers in order to increase system performance. Finally, this work suggests considering behavior topics in security questions that have sufficient details to ensure that users remember their responses while others would find responses difficult to predict.

## Supplemental Information

10.7717/peerj-cs.903/supp-1 Supplemental Information 1 Raw data

10.7717/peerj-cs.903/supp-2 Supplemental Information 2 Questionnaire

## Additional Information and Declarations

### Data Availability

The following information was supplied regarding data availability: The raw data are available in the Supplemental File.
