# Supplementary material for: Evaluating knowledge-based security questions for fallback authentication
Source: PeerJ Comput Sci. 2022 Mar 11;8:e903. doi: 10.7717/peerj-cs.903 (PMC9044221; doi:10.7717/peerj-cs.903)
Supplement: Supplemental Information 2 [file peerj-cs-08-903-s002.pdf]

Project description

This study explores the direction of fallback authentication research, knowledge gaps, and areas of future work through a systematic literature review. In addition, this study aims to improve security and usability of fallback authentication focusing on static security questions. The security and usability of static security questions are analysing in this study through conducting online experiment spanned in two part. The study aims to explore different objectives. First, the study explores the effect of question features. Second, the study explores the effect of using supporting methods to compose memorable answers. In addition, this study investigates the effect of following user guidance in composing secure answers .

The approach in this study will be quantitative by an experiment survey. The population of the study will be different of the demographic details (gender, technical background, educational, and method of creating and saving knowledge-based authentication data). The experiment survey structured to be in multi languages language (English and Arabic) to be accessible by all audience. The link of the survey will be available online to participant and they can fill it in any time. The time spent in each question will be recorded, in order to measure usability feature. Participation will be voluntary by email message; the data will be analysed using the Security Model Testing and different methods of usability testing. This study used google Apps script to create the experiment survey.

The Evaluation of Fallback Authentication

Email Message

Dear Participant,

You are being asked to volunteer in a research study, 'Study on Fallback Authentication Methods'. The purpose of the study is to explores effected factors on user authentication by security challenges questions. If you agree to participate, your part will be to complete:

Experiment 1: a short experiment and several simple questions.
Experiment 2: after 15 days, Experiment 1 will be repeat in order to measure your memorability of your answer.
The experiment parts should take no more than 20 minutes of your time.
Your User ID is:
(For the purpose of comparing your answers in Experiment 1 and Experiment 2)

The link of Experiment 1: Experiment 1
To enable a clear screen view, it is recommended to open the link by computer browsers.

If you have any questions contact Reem AlHusain, e-mail: 391214359@qu.edu.sa

Thank you,
Research team

Survey Questionnaire

Please fill the following fields

الرجاء تكملة البيانات التالية

User-Id (رقم المشارك)	--
Email optional (البريد الالكتروني اختياري)	--
Age(العمر)	○ 18-23<br>○ 24-34<br>○ 35-45<br>○ 50 or older
Gender(الجنس)	○ Male (ذكر)<br>○ Female (انثى)
Education(التعليم)	○ High school (الثانوية)<br>○ Diploma (دبلوم)<br>○ Bachelor (بكالوريوس)<br>○ Master degree(ماجستير)<br>○ Ph.D. degree(دكتوراه)<br>○ Other(اخرى)
Technical background (الخلفية التقنية)	○ No background(لا توجد خلفية)<br>○ Medium background(خلفية متوسطة)<br>○ High background or specialist خلفية)<br>عالية او متخصص)
You select passwords and answers to the security questions based on:<br>طريقتك بإنشاء كلمات المرور واجابات اسئلة الامان)<br>بالاعتماد على:)	○ Randomly way(بطريقة عشوائية)<br>Includes specifics names بتضمين)<br>كلمات وأسماء معينة)<br>○ Includes specific dates e.g., birthdate بتضمين تواريخ محددة مثل)<br>تاريخ الميلاد)<br>○ Complex words (كلمات معقدة)
Your method to vave authentication data<br>(طريقتك بحفظ بيانات المصادقة مثل كلمات<br>المرور والاجابات)	○ By memorize only الاعتماد على)<br>الذاكرة والحفظ فقط)<br>○ By automatic login and credentials saving الاعتماد على الدخول التلقائي وحفظ)<br>بيانات الدخول)<br>○ By written dawn(عن طريق تدوينها)

	○ By memorize and using helping methods e.g., shortcuts and your own code (التذكر مع استخدام طرق مساعدة مثل التلميحات)
ملاحظة:<br><br>الجزء التالي يتضمن التجربة التطبيقية، اتبع التعليمات المرفقة بكل صفحة ومراجعة اجاباتك للسؤال قبل الانتقال للصفحة التالية<br><br>علما ان الرجوع للصفحة السابقة غير متاح وهذا بهدف قياس عامل الوقت المستغرق<br><br>اضافة الى ذلك يجب التذكير بان هذا هو الجزء الاول من التجربة وبعد فترة سيتم اعادة هذه التجربة لاختبار مدى تذكر المستخدم للإجابات<br><br>مع الحرص على عدم الاحتفاظ بنص الاجابة بأي وسيلة وهذا بهدف قياس عامل التذكر<br><br>احاطة بأن جميع البيانات سوف تستخدم لغرض الدراسة فقط<br><br>جزيل الشكر	
Note:<br><br>The next part includes the experience<br><br>Follow the instructions attached to each page and review your answers to the question before moving to the next page<br><br>Note that: returning to the previous page is not available, and this is in order to measure the time spent factor<br><br>In addition, it must be reminded that this is the first part of the experiment, and after a while, this experiment will be repeated to test the user's memorized of the answers<br><br>By considering of not keep the plain text of the answers in any way, this is in order to measure the memorability factor<br><br>Note that all data will be used for the purpose of the study only<br><br>Thanks,	

Part1:
الجزء الأول

Section one contains: (Q1 and Q2), try to answer them by a strong and memorable answer,

الجزء الأول يتضمن:(السؤال الأول والثاني)، اختر اجابة آمنة وسهلة التذكر بالنسبة لك

<u>The goal in this section (measure your memory level)</u>

<u>الهدف من الجزء الأول هو قياس مستوى التذكر</u>

**Q1.1 From the list below, select your favorite class in high school**<br><br>**اختر من القائمة المادة المفضلة لك بمرحلة الثانوية**	○ Arabic اللغة العربية<br>○ Math الرياضيات<br>○ Geography الجغرافيا<br>○ Chemistry الكيمياء<br>○ Computer Science علوم الحاسب<br>○ Physics الفيزياء<br>○ English اللغة الانجليزية<br>○ History التاريخ<br>○ Biology علم طبقات الارض<br>○ Psychology and Sociology علم النفس والاجتماع
**Q1.2 What was your teacher's name of this course?**<br><br>**ماذا كان اسم مدرس تلك المادة**	---

**Q2 Your dream job or business is ______?**<br><br>**وظيفة او مشروع احلامك هو ______**	--

Part2:
الجزء الثاني

Section two contains four question (Q3&Q4&Q5&Q6). These question you have to answer them by using aid tools. You can find a complete description of the aid tools below:

الجزء الثاني يتضمن اربعة أسئلة (سؤال ٣&٤&٥&٦)

يجب الاجابة على هذه الاسئلة باستخدام وسيلة مساعدة، توضيح كامل للطرق المساعدة يمكنك الاطلاع عليه بالأسفل

click here

The goal in this section (measure the effect of aid tools in composing strong and memorable answer)
الهدف من الجزء الثاني من التجربة هو معرفة تأثير استخدام الطرق المساعدة على تكوين اجابات آمنة وسهلة التذكر

Note that:

- ✓ **You should not use the same examples and/or formulas given in the above methods You should create your own formula and steps.**
- ✓ **You can apply your formula to answer all questions in section 2, which contains a note of must use aid tool to answering.**
- ✓ **You should never share your formula with anyone.**

ملاحظة:

- ✓ **الوصف المقدم للطرق المساعدة يعطي لمحة عامه عن الطريقة ولا ينبغي تطبيق نفس القاعدة او الأمثلة بل يجب على المستخدم تكوين قاعدته الخاصة بعدد خطوات حسب اختياره لكن بنفس المفهوم**
- ✓ **بالإمكان تطبيق القاعدة المختارة للإجابة على جميع أسئلة الجزء الثاني وموضح عند كل سؤال انه يجب الاجابة بالاعتماد على طريقة مساعدة**
- ✓ **عدم مشاركة هذه القاعدة مع أي أحد او كتابتها والاحتفاظ بها**

**Q3 After an achievement in your work, name the favorite vacation place?**<br>**ما هو اسم مكان الاجازة المفضل لك بعد تحقيقك لإنجاز في العمل؟**<br><br>***Must apply aid tool to answer**<br>**ملاحظة: يجب الاجابة بالاعتماد على طريقة مساعدة ***	--

**Q4 Write a description of the the image below**<br>**أوصف الصورة ادناه**<br><br>***Must apply aid tool to answer**<br>**ملاحظة: يجب الاجابة بالاعتماد على طريقة مساعدة ***	--

**Q5 What is your favorite mobile applications?**<br>**ماهي تطبيقات هاتفك المفضلة**<br>***Must apply aid tool to answer**<br>**ملاحظة: يجب الاجابة بالاعتماد على طريقة مساعدة ***<br><br>**1. Your answer must be at least 8 characters long**<br>**2. Your answer must contain at least one upper case, one lower case, one number and one special keyboard character.**<br>**3. Your answer should not contain personal information such as name, id, and date of birth.**<br>**4. Your answer should not be easily guessable**<br>**5. once you start typing your answer the text background will become on red color and after fulfill a condition 1 and 2 text background will back to a white color**<br><br>**فضلاً تأكد ان الإجابة تحقق الشروط التالية:**<br>**أولاً**: أن يبلغ طولها ثمانية أحرف على الأقل<br>**ثانياً:** ان تتكون من أحرف كبيرة وصغيرة وارقام ورموز<br>**ثالثاً**: ألا تحتوي على معلومات شخصية ويمكن معرفتها من قبل الاخرين مثل الاسماء وتاريخ الميلاد<br>**رابعاً**: يجب ألا تكون الاجابة بسيطة وسهلة التخمين<br>**خامساً**: عند البدء بالإدخال خلفية مربع النص ستتغير للون الاحمر وتعود للون الابيض منذ تحقيق الشرطين الاول والثاني اعلاه	--

**Q6 Select an English words that you usually pronounced correctly?**<br>**اختر مجموعة كلمات من اللغة الانجليزية التي دائما تتقن نطقها**	

***Must apply aid tool to answer**<br>**ملاحظة: يجب الاجابة بالاعتماد على طريقة مساعدة ***<br><br>1. **Your answer must be at least 8 characters long**<br>2. **Your answer must contain at least one upper case, one lower case, one number and one special keyboard character.**<br>3. **Your answer should not contain personal information such as name, id, and date of birth.**<br>4. **Your answer should not be easily guessable**<br>5. **once you start typing your answer the text background will become on red color and after fulfill a condition 1 and 2 text background will back to a white color**<br><br>**فضلاً تأكد ان الإجابة تحقق الشروط التالية:**<br>**أولاً**: أن يبلغ طولها ثمانية أحرف على الأقل<br>**ثانياً**: ان تتكون من أحرف كبيرة وصغيرة وارقام ورموز<br>**ثالثاً**: ألا تحتوي على معلومات شخصية ويمكن معرفتها من قبل الاخرين مثل الاسماء وتاريخ الميلاد<br>**رابعاً**: يجب ألا تكون الاجابة بسيطة وسهلة التخمين<br>**خامساً**: عند البدء بالإدخال خلفية مربع النص ستتغير للون الاحمر وتعود للون الابيض منذ تحقيق الشرطين الاول والثاني اعلاه	--

Part3:
الجزء الثالث

Section three contains four questions ،just select the answer that you think can remember it later.
الجزء الثالث يحتوي على اربعة اسئلة، فقط عليك اختيار الاجابة المناسبة لك والتي باعتقادك يمكنك تذكرها لاحقاً

The goal of this section measures the effect of recognition mechanism in graphics based and behavior-based questions
الهدف من الجزء الثالث معرفة تأثير استخدام الاختيارات المبنية على الجرافيك وسلوك المستخدم

<table>
<tr><td>Q7 From the list below, select the image that reflects your hobby
اختار الصورة التي تعكس هوايتك من القائمة ادناه
<table>
<tr><td>Image 1</td><td>Image 2</td><td>Image 3</td></tr>
<tr><td>Image 4</td><td>Image 5</td><td>Image 6</td></tr>
<tr><td>Image 7</td><td>Image 8</td><td>Image 9</td></tr>
<tr><td>Image 10</td><td>Image 11</td><td>Image 12</td></tr>
</table></td>
<td>○ Image 1
○ Image 2
○ Image 3
○ Image 4
○ Image 5
○ Image 6
○ Image 7
○ Image 8
○ Image 9
○ Image 10
○ Image 11
○ Image 12</td></tr>
</table>

**Q8 IF Your national ID Expiration date is in this year, in which of the available options you may select to renew it?**<br><br>**إذا كانت هويتك الوطنية تتطلب تجديد هذه السنة، خلال اي فترة قبل يوم الانتهاء ستفكر بالقيام باصدار نسخة جديدة**	○ **Before 6 months (قبل ٦ أشهر)**<br>○ **Before 3 months (قبل ٣ أشهر)**<br>○ **Before 2 months (قبل شهرين)**<br>○ **Before 1 month (قبل شهر)**<br>○ **Before 2 weeks (قبل اسبوعين)**<br>○ **Before 1 weeks (قبل اسبوع)**

**Q9 What was the color of your first mobile phone?**<br><br>**ماذا كان لون اول هاتف محمول لك**	**Color picker option**<br>**خيار تحديد لون**

**Q10 In case you invited to attend a formal event, what the most thing you will think of?**<br><br>**في حال تم دعوتك لحضور مناسبة رسمية ما هو أكثر شيء سيشغل تفكيرك**	○ **Preparation to arrive at the exact time (الحضور بالوقت المحدد)**<br>○ **Thinking about your wearing style and selection (الاستعداد وتنسيق مظهرك الخارجي)**<br>○ **Exited to meet people and conversation (مقابلة الحضور وتبادل النقاش)**<br>○ **Think about the time you will spend (مدة المناسبة والوقت المستغرق)**

Participant evaluation
(تقييم المشارك)

**Thank you so much for your participant, we truly appreciate your support.Finally, we are happy to know your feedback by answering the following points:**<br><br>**شكرا جزيلا لمشاركتك، نقدر مساهمتك في الدراسة ودعمك. ويسعدنا معرفة كيف كانت تجربتك من خلال تقييم النقاط التالية:**

	Strongly agree موافق بشدة	Agree موافق	Neutral محايد	Dissagree غير موافق	Strongly dissagree غير موافق بشدة
**The level of recall answer in questions of section three (the choicess) is high**<br><br>**مستوى تذكرك اجابة اسئلة الجزء الثالث عالي (الاسئلة الاختيارية)**	○	○	○	○	○
**The aid tools had a positive effect on memorizing your answers**<br><br>**الطرق المساعدة لها تأثير ايجابي على تذكرك للإجابات**	○	○	○	○	○
**The answer creation based on aid tools is safe and secure**<br><br>**الاجابة باستخدام الوسائل المساعدة هي اجابة آمنه ومضمونة**	○	○	○	○	○
**Applying aid tools method was convenient**<br><br>**كان تطبيق الوسائل المساعدة مريح ومناسب**	○	○	○	○	○
**This method of answer based on aid tools was trustworthy**<br><br>**كان اعتماد طريقة الوسائل المساعدة جدير بالثقة**	○	○	○	○	○
**In the future, you intend to apply the aid tools in creating knowledge-based authentication data e.g., passwords, and security answer**<br><br>**مستقبلاً تنوي اعتماد طريقة الوسائل المساعدة في تكوين بيانات المصادقة المعتمدة على عامل المعرفة مثل كلمات المرور واجابات اسئلة الامان**	○	○	○	○	○
**Additional feedback**<br><br>**ملاحظات اضافية**					
